# Impact of sickness induced by centrifugation on tilt perception

**DOI:** 10.3389/fneur.2025.1628938

**Published:** 2025-08-13

**Authors:** Taylor L. Lonner, Caroline R. Austin, Joanna S. Blake, Parinie Gupta, Jason M. Katz, Aadhit R. Gopinath, Torin K. Clark

**Affiliations:** Smead Department of Aerospace Engineering Sciences, University of Colorado Boulder, Boulder, CO, United States

**Keywords:** centrifugation, vestibular, orientation perception, spatial disorientation, sickness induced by centrifugation

## Abstract

**Introduction:**

Sickness induced by centrifugation (SIC) is an analog for sensorimotor impairment and motion sickness associated with gravity transitions experienced by astronauts. The paradigm involves sustained centrifugation to create a static Gx (into the eyes) hypergravity exposure, following which vestibular-mediated functions, such as balance and eye movements, have been found to be degraded or altered. Furthermore, astronauts who were more prone to space motion sickness were also more susceptible to motion sickness following SIC. However, the vestibular and perceptual processing alterations induced by SIC remain poorly understood as human tilt perception following SIC has not yet been quantified.

**Methods:**

We assessed the impact of SIC on the perception of self-roll tilt and pitch tilt in a total of twenty healthy subjects. On one testing day, the subjects were exposed to the SIC analog, wherein they underwent an hour of 2Gx centrifugation. Afterwards, they reported tilt perception while seated in the dark during a variety of static and dynamic tilt and translation motion profiles, either in a roll tilt or pitch tilt configuration. These results were compared to tilt perception following a baseline condition on a separate testing day where subjects laid supine for an hour.

**Results:**

When compared to the baseline condition, SIC exposure resulted in a significant underestimation of −33.2% in the pitch tilt angle (*t*(60) = −4.39, *p* < 0.0005), but no effect in roll (mean underestimation of −7.5%, *t*(60) = −0.68, *p* = 0.50).

**Discussion:**

We discuss the implications of these vestibular perceptual effects of SIC as an analog for spaceflight-associated spatial disorientation.

## Introduction

Spatial disorientation (SD)—defined as a failure in correctly sensing “the position, motion, or attitude of [an] aircraft or [oneself] within [a] fixed coordinate system” (1)—is often caused by a heightened reliance on the vestibular system for orientation within degraded visual environments (DVEs) such as nighttime flying, flying through clouds, or brownouts during landings. The vestibular system consists of two sets of organs in each inner ear: otoliths that sense linear acceleration and semicircular canals that sense angular velocity. In the absence of visual information, the signals from these organs can be misinterpreted by the central nervous system due to limitations of the physical sensors. The otoliths can only sense the net combination of all linear accelerations, meaning that during coordinated turns (where the net force vector aligns with the body axis) or during translations, the signals sent from the otoliths may result in a misperception of the ‘down’ direction. Additionally, given the mechanical properties of the semicircular canals, a constant angular velocity results in a decaying signal over time. Without visual input, this can be interpreted by the central nervous system as a slowing in rotation, and upon stopping, can result in the sensation of turning in the opposite direction (1, 2).

For spaceflight, in addition to DVEs, there is a concern that gravity transitions will significantly impair vestibular perception, exacerbating these misperceptions and endangering pilots during landing operations on the Moon or other surface environments. Following gravity transitions, the central nervous system must adapt to a new gravity environment, adjusting its interpretation of signals from sensory peripherals to better align with reality (3, 4). During adaptation to microgravity, astronauts have mentioned experiencing illusory sensations of motion and incorrect perceptions of orientation (5, 6). Furthermore, transitions to altered gravity environments, such as hypogravity or hypergravity, have resulted in the underestimation and overestimation of tilt perceptions, respectively, in what is known as the tilt-gain illusion (7–9). Finally, astronauts returning to Earth from microgravity have described a series of tilt-translation illusions wherein head tilts would result in illusory sensations of translation (10–14).

To date, these illusions have been predominantly researched in microgravity environments, such as on the International Space Station or during parabolic flights. As such, research and development of potential countermeasure systems for astronaut SD are stifled by long timeframes, small sample sizes, and expenses arising from microgravity studies. Therefore, it is of interest to the scientific community to investigate ground-based analogs for researching the effects of gravity transitions on orientation perception. One such analog with promise for recreating various dysfunctions mimicking those following spaceflight is the Sickness Induced by Centrifugation paradigm.

The Sickness Induced by Centrifugation (SIC) (15–24) paradigm has been employed to recreate the effects of a gravity transition relevant to spatial disorientation in this study. This paradigm posits that extended exposure to hypergravity via centrifugation, followed by a return to Earth’s gravity, can recreate the symptoms astronauts experience when initially exposed to microgravity. The direction of hypergravity exposure is typically in the head-centered *x*-axis due to its better cardiovascular tolerability and comfort level at high gravitational loading magnitudes over longer durations (25).

Historically, this analog has been used to reproduce symptomologies analogous to Space Motion Sickness (SMS) (22, 24). More recently, it has also been employed as an analog for Terrestrial Readaptation Motion Sickness (TRMS), also called Entry Motion Sickness (EMS), when returning to Earth after extended adaptation to microgravity (26, 27). Studies have also found more general sensorimotor decrements following SIC, such as balance decline (15, 20, 24, 26), reductions in ocular torsion (19), and qualitatively reported illusory sensations of motion (20, 28), suggesting that it may be a valuable tool for inducing relevant vestibular perceptual changes following gravity transitions. However, changes in spatial orientation perception following SIC have not yet been quantified, creating a gap in our understanding of the vestibular and perceptual changes induced by SIC and how those are related to gravity transitions experienced in spaceflight.

## Methods

To determine the impact of SIC on tilt perception in roll and pitch, two datasets from separate participant cohorts were collected. On two separate testing days in a within-subject design, participants were exposed to different gravity environments prior to performing a continuous Subjective Haptic Horizontal (SHH) task to report self-tilt perception during a set of dynamic and static head-centered roll or pitch tilts. Testing occurred over two separate days—rather than testing before and after SIC exposure—to reduce ordering effects on the SHH task, such as learning or fatigue, and to control for additional confounds such as visuals prior to the SHH task, ambulation, or head movements. The SHH task involved the participant continuously tilting a hollow square aluminum bar in roll or an aluminum plate in pitch to keep it level with their perception of Earth-fixed horizontal as they underwent tilts in those respective axes (29, 30).

The SHH has been used extensively in the literature as a measure of tilt perception (31–33). For the roll-tilt experiment, the bar was mounted in front of the participant, who was instructed to lightly hold both edges of the bar. A potentiometer (Vishay Spectrol 601 HE) was mounted at the center of the bar to record its angle during motion. For pitch, the plate was mounted on the right side of the chair, and the participants were instructed to rest their right palm on the plate. The same potentiometer was attached to the center of the plate to determine the pitch angle of the plate during motion.

### Gravity protocols

On each testing day, the participants experienced a specific gravity protocol: Supine (control) or SIC (experimental). The order of the testing sessions was counterbalanced across the participant cohort to protect against confounding effects of ordering (learning, fatigue, etc.) and performed on separate days. The gravity protocol for the Supine session consisted of participants lying supine for one hour on a padded table with their head straight such that the resultant gravity vector was in the negative head-centered *x*-axis (into the eyes).

On the SIC testing day, participants underwent an hour of 2Gx combined centrifugal and gravitational force using SIC, where the net force was in the same head-centered direction (into the eyes). SIC was applied using the Human Eccentric Rotator Device (HERD) at the University of Colorado, Boulder (Supplementary Figures 1a,b). This nine-foot short-radius centrifuge consists of a padded bed on one end tilted back 30° from the vertical with thick cushioning on both sides of the participant’s head to discourage head tilts while rotating, thereby preventing Coriolis cross-coupling during centrifugation (34, 35). The HERD spun participants at 24 rotations per minute (RPM) to generate 2Gs of force at the head—the safety-tested limit of the device. Participants were secured using a five-point harness. To ensure that 24 RPM would generate 2Gs at the head, an adjustable footplate on the bed could be raised or lowered to maintain the same head position across all participants.

While spinning, the participants had two-way audio communication with the operators, and a one-way video was available for the operators to monitor the participants. Acceleration to 24 RPM was done gradually to ensure participant comfort, first taking 120 seconds to reach 10 RPM before incrementing up to 24 RPM at an average rate of 10 seconds/RPM when the participant indicated that they were ready. As the device accelerated and the direction of the net force vector rotated, participants reliably commented on the sensation of slowly tilting backward. Handholds were provided to minimize the participants’ illusion of instability. The spin-down occurred over 120 seconds. Throughout the session, the participants had access to two emergency stop mechanisms: a traditional red button and a secondary seatbelt.

In both conditions, the participants were in the dark, not ambulating, and asked to keep their heads still. Thus, the primary difference was that SIC exposed subjects to 2Gx, while Supine was only 1Gx. Every five min, participants were tasked with verbally reporting their motion sickness using the Fast Motion Sickness Scale (FMS), where on a 0–20 scale, 20 was vomiting and 10 was halfway to vomiting (36), as well as their general discomfort on a scale of ‘none,’ ‘slight,’ ‘moderate,’ and ‘severe’.

### Tilt perception profiles

**Figure 1:**
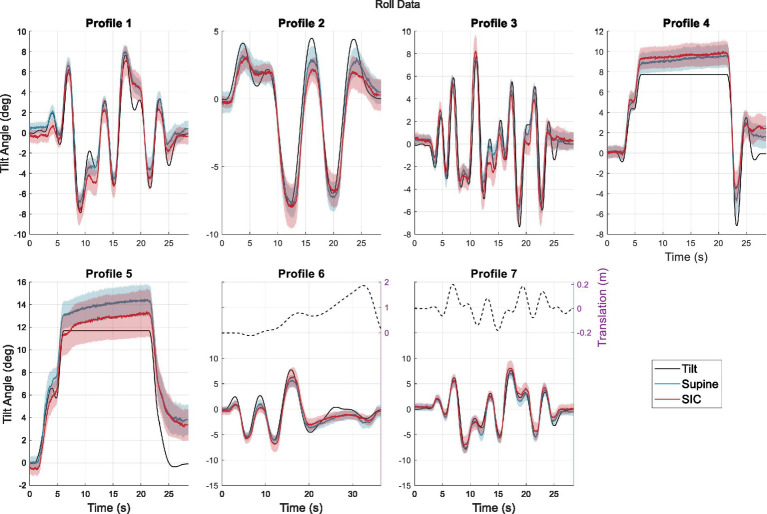
Average perceptions across participants during roll tilt. The solid black line indicates the true tilt, while the blue line shows perception following the Supine condition, and the red line shows perception following the SIC condition. The shaded regions indicate standard errors. The profiles consisted of sum-of-sine waves with low (< 0.15 Hz: Profiles 2 and 6), medium (0.07–0.31 Hz: Profiles 1 and 7), and high (0.2–0.47 Hz: Profile 3) frequency contents, as well as two static profiles (Profiles 4 and 5). For dynamic profiles, the maximum tilt amplitude was 8°. Static Profile 5 reaches 12°. Profiles 6 and 7 also contain lateral translation profiles (dashed line).

The motion profiles for the SHH task (Figure 1) were performed using the Tilt Translation Sled (TTS) at the University of Colorado, Boulder: a two-degree-of-freedom (DOF) motion device capable of either roll tilt and lateral translation or pitch tilt and fore-aft translation (Supplementary Figures 1c,d). It was used in both configurations throughout this study with two unique testing cohorts. Profiles were generated to encapsulate a variety of tilt and tilt-with-translation profiles to explore specific processing pathways in vestibular systems. This included (Figure 1) pseudo-random sum of sines dynamic tilt profiles at moderate (Profile 1), slower (2), and faster (3) frequencies; static tilt profiles at smaller (4) and larger (5) angles; and dynamic profiles that included concurrent translation to alter the net gravito-inertial stimulation to the otoliths (6 and 7). In the analysis, we considered the effect of these seven unique motion profiles, as well as whether the effects of SIC vs. Supine differed across motion profiles. We also considered grouping the motion profiles by their frequency content, since higher frequency angular rotations are better transduced by the semicircular canals, and we anticipated that this might differentiate how SIC impacted tilt perception. Specifically, we grouped the motion profiles into static tilt (Profiles 4 and 5), low frequency (Profiles 2 and 6), and high frequency (Profiles 1, 3, and 7) for further analysis to reduce extra degrees of freedom in the statistical models for all seven motion profiles.

The tilt perception protocol consisted of two blocks of profiles. Within a block, each profile was run in the positive direction (shown in Figure 1, where positive tilts are right ear down for roll and forward or nose-down in pitch) as well as inverted to account for any directional biases. The order of the 14 profiles was randomized within each block, resulting in a total of 28 profiles across both blocks, and the subjects remained naïve to the motion profiles being performed or repeated.

While in the TTS, participants were seated and secured using a five-point harness and head restraint. Again, participants had access to an emergency stop mechanism. During the trials, the participants were in the dark with their eyes closed; lights were turned on to indicate the end of a trial. Two-way audio and one-way video were available to the operators, allowing communication and monitoring of the participants within the TTS. The participants were reminded of the SHH task every four trials and were also informed that the reminder was not a reflection of their performance during the trials. The FMS was administered every two trials to assess the participants’ wellbeing and tolerance to motion.

### Experimental design

In the first testing session, participants were introduced to the two motion devices and given the option of seeing the devices in motion via recorded videos before agreeing to participate in the study. Prior to any gravity exposure protocols, the participants received training on the SHH task in the TTS. They underwent at least two training trials to familiarize themselves with the SHH tool and were allowed to continue practicing until they were comfortable and confident in the task, and the operators could confirm that they were moving the SHH devices in the appropriate direction. During this training session, the operators provided general feedback regarding the correct or incorrect use of the SHH tool. Once both parties were satisfied with the participant’s performance, the participant was removed from the TTS and brought to their first gravity protocol.

Following either hour of gravity exposure, participants were transferred back into the TTS via a wheelchair with their heads restrained to delay readaptation to Earth gravity and prevent head-tilt-induced vertigo resulting from SIC. This transfer (from finishing the gravity protocol to starting the first tilt perception trial) took approximately 11 min for 2G gravity exposure and 7 min for the 1G condition. During the SHH tilt perception task in the TTS, participants were given short breaks between trials before being asked if they were ready to continue the task. The 28 profiles took approximately 30 min to complete. The subsequent testing session was separated by at least two and no more than 6 days to allow any potential SIC carry-over effects to subside (if that was the first session) while ensuring the participant did not forget how to perform the SHH task.

### SHH processing

Raw data from the SHH task (i.e., deflection angle of the haptic bar or plate over time during each motion profile) was processed in a similar manner as has been done previously (29) to produce a cleaned measure of tilt perception over time. Specifically, data-driven adjustments were made for the bias, time delay, and gain. One bias correction was calculated for each participant on each testing day to account for potential changes in the installation of the SHH bar or plate, slight shifts in the homing of the chair, and interpersonal differences in perceived upright. The time average of the SHH angle for each dynamic trial was calculated and compared with the time average of the actual tilt profile. The mean bias across all the trials and profiles for each subject on each testing day was used to offset the SHH data for the corresponding subject and testing day. These values ranged from −2.14° to 1.66° in roll and −5.60° to 0.86° in pitch. In pitch, a negative bias corresponds to a participant tilting the plate backward more than the chair was pitched forward.

Following this bias correction, a time delay correction was applied to account for delays in the response rate. For each trial on each testing day, a grid search was performed between 0 and 1 s to determine which time delay best aligned the SHH data with the tilt profiles. The median time delay was computed for both days, ranging from 0.1 to 0.5 s in roll and 0.02 to 0.54 s in pitch, and was applied to align the stimulus and response measures in time along the *x*-axis between the SHH data and tilt profile.

Having adjusted for any bias and slight time delays, a gain correction was calculated from the data collected only on the Supine testing day, since we anticipated generally accurate perceptions of tilt in the control condition. Another grid search was performed to determine the multiplicative gain that minimized the average difference between the SHH data and tilt profile. Each subject’s mean gain adjustment across trials and profiles following Supine was then applied to data from the corresponding subject’s data on both testing days to enable a comparison of over- or underestimation between Supine and SIC. These gain adjustment values ranged from 0.59 to 5.54 in roll and 0.63 to 5.19 in pitch (values greater than 1 correspond to the participant deflecting the SHH bar/plate less than they were actually being tilted). Supplementary Figures 2, 3 show visualizations of these biases, time delays, and gain adjustments for each subject.

Occasionally, subjects would become confused or make a mistake while performing the SHH task in a manner that we believe was not representative of their underlying tilt perception. We identified those trials with strict criteria and excluded them from subsequent analyses. Two independent judges, blinded to the condition (SIC or Supine), reviewed plots of the adjusted SHH over time and estimated the proportion of time in which non-perceptual reporting (i.e., moving the bar/plate in the opposite direction, non-reporting, or otherwise distracted motions) occurred. If the average across judges exceeded 10 seconds, it was deemed to substantially impact the trial’s perceptual outcome and was excluded. Additionally, trials where the linear relationship between the uncorrected reported tilt and the actual tilt was negative (i.e., the participant likely inverted the direction in which they were supposed to deflect the SHH bar/plate) were removed. No data were removed from the roll tilt dataset (from 560 trials, 28 per SIC and Supine, for each of the 10 subjects), and 31 trials were omitted from the pitch tilt dataset (out of 560), accounting for 5.5% of the dataset. Of these 31 trials, five were from the Supine testing day and 26 were following SIC, suggesting that SIC may have led to more confusion in the SHH reporting task during pitch tilt. All profiles had some data removed, with a minimum of three removed from Profile 3 and a maximum of seven removed from Profile 7.

**Figure 2:**
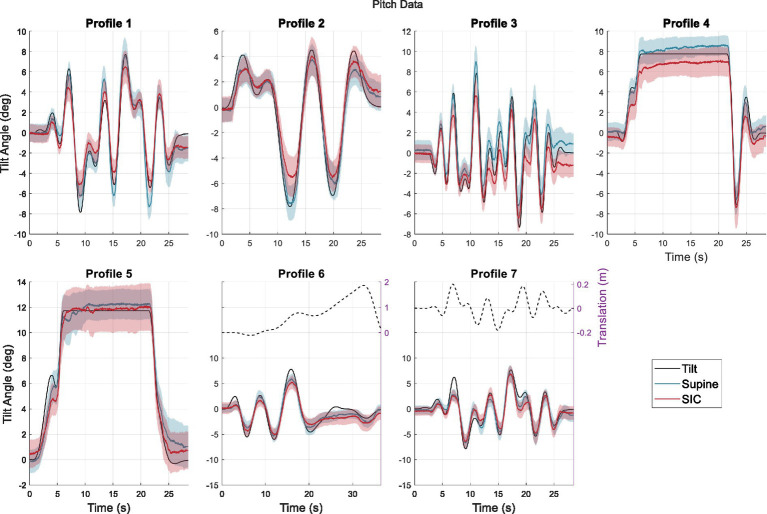
Average perceptions across participants during pitch tilt. The solid black line indicates the true tilt, while the blue line shows perception following the Supine condition, and the red line shows perception following the SIC condition. Shaded regions indicate standard errors. The dashed black lines are translation motions in the fore-aft direction for the profiles that contain them.

Adjusted SHH data across the four trials each participant underwent for each profile on each testing session was averaged across trials to produce a “best” measure of participant perception for every profile and gravity protocol (the inverted replicate responses were multiplied by −1 before being averaged with the positive-profile responses). These best approximations were then averaged across participants to produce group perceptions and standard errors in both roll (Figure 1) and pitch (Figure 2).

### Perceptual metrics and statistical analyses

We computed two metrics to capture the overall under- or overestimation of tilt perception. The first metric compares perception post-SIC to perception post-Supine as a percent difference. The second, instead, compares perception (either post-SIC or post-supine) to the actual tilt, providing context relative to the actual tilt (29). Further details on how each of these metrics is computed are provided here.

The simplest measure of perceptual differences is the percent difference from the post-Supine condition using a participant’s “best” measure of perception. Within a profile, at every time point, the percent difference from the post-Supine condition was calculated by examining the difference between tilt perception following SIC against Supine and normalizing it by post-Supine perception. This would produce a time series of percent differences from Supine, where positive values are moments of overestimation and negative values are moments of underestimation with respect to the Supine condition. At small angles, these values can become exceedingly large; therefore, the median over the time series is selected when the motion profile absolute tilt values are larger than 0.5° to avoid artificial inflation in either direction.

The next metric is a direct measurement of the linear relationship between the SHH task data points and the actual tilt profile, hereafter referred to as the perception slope. The “best” measure of perception for each subject on a given profile and testing day was plotted against the tilt motion and fitted using a linear function. The slope was extracted as a metric. Here, perfect perception is represented by unity, underestimation is less than one, and overestimation is greater than one (Supplementary Figure 4).

These continuous metrics were analyzed using R (version 4.4.1; RStudio 2024.04.02) to characterize the participants’ tilt perception during SHH tasks. Parametric statistics were employed using data transformations where necessary. The tests included repeated-measures analyses of variance (RMANOVA), linear mixed-effects models, and Student’s t-tests. Parametric assumptions, such as normality, continuity, and homoscedasticity, were verified for relevant statistical tests.

### Participants

A total of 22 individuals signed the informed consent form to participate in the study, of which two participants voluntarily withdrew prior to completing both testing sessions and were therefore not included in the analysis. Subjects completed the short-form Motion Sickness Susceptibility (MSSQ) form to gauge their percentile of susceptibility, as well as questionnaires on demographics and piloting experience. The remaining participants were split between roll perception (*n* = 10, female = 2, age = 23.1±2.9 , MSSQ = 25.6±15.0 %) and pitch perception (*n* = 10, female = 4, age = 23.3±4.1 , MSSQ = 18.1±16.1 %). In both experimental groups, there were three participants with piloting experience. All participants were right-hand dominant, except for one subject in the pitch cohort. Of the 20 subjects who participated, 13 self-identified as White and non-Hispanic/Latino, two self-identified as White and Hispanic/Latino, four self-identified as Asian, and one self-identified as Middle Eastern/North African.

All participants signed a written informed consent prior to participating in the study. The study was approved by the Institutional Review Board of the University of Colorado, Boulder (Protocol number 24–0157) in accordance with the Declaration of Helsinki.

## Results

Figures 1, 2 visualize the average perceptions of roll tilt and pitch tilt, respectively, during the various motion profiles for roll and pitch. In these figures, the blue lines indicate group perception following the Supine protocol, while the red lines indicate group perception for the same motion profiles following the SIC protocol. The black lines show the actual motion profiles (solid for tilt and dashed for translation, where applicable). In previous studies using SIC, the results indicated that the effects of SIC may be relatively short-lived (20). To identify any readaptation effects, the average perception after the first block of trials was initially compared to that of the second block of trials. However, no significant differences were identified, suggesting that readaptation had not occurred within 40 min following SIC, and we could reasonably average across all trials (Supplementary Figures 5, 6).

Figures 1, 2 demonstrate general group trends toward perceptual accuracy and differences following SIC. In roll, the differences between SIC and Supine were less obvious, with a notable exception during Profile 5, where the average post-SIC perception was smaller than the average post-Supine perception. Conversely, there was a more noticeable and consistent effect in response to pitch tilt. In Profile 4, post-SIC perception was smaller than post-Supine perception, and this trend can also be noticed at the motion peaks in the dynamic profiles as well.

**Figure 3:**
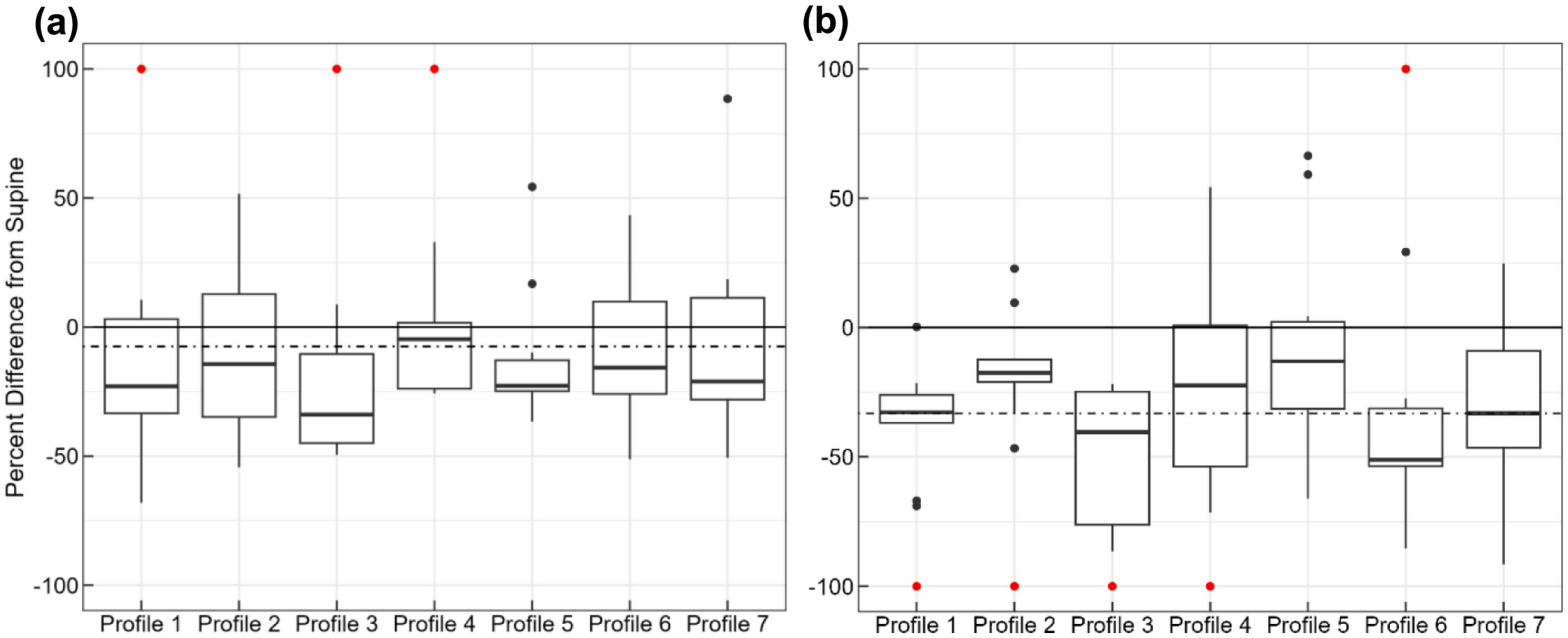
Percent difference between post-SIC and post-Supine tilt perception in both roll **(a)** and pitch **(b)** across the seven unique profiles, where positive values correspond to overestimations following SIC in comparison to Supine. The solid black line indicates a 0 % difference, and the dashed line indicates the average percentage difference across participants and profiles. There are ten data points in each box. Red data points indicate outliers that extend past ± 100%.

To quantify the under- (or over-) estimation in perceived tilt following SIC compared to Supine, we computed the percent difference (as a median of the time points over each trial). Positive values corresponded to the overestimation of tilt following SIC compared to that after the Supine position, while negative values implied the underestimation of tilt after SIC. In general, the distributions of this metric across profiles fell below zero (Figure 3), corresponding to smaller perceptions of tilt following SIC as compared to the control condition following Supine. First, we considered whether this metric varied across the seven different motion profiles or was affected by the available demographic measures. A one-way ANOVA with repeated measures across profiles was performed to determine the effect of the profile on this percent difference (after running tests that identified that none of the collected demographic information was significant). For both roll and pitch, the effect of the motion profiles failed to reach significance (*F*_roll_(6, 54) = 0.95, *p*_roll_ = 0.47; *F*_pitch_(6, 54) = 0.94, *p*_pitch_ = 0.09). Although we cannot conclude that the seven motion profiles had no effect, the insignificant statistical result suggests that the effects of SIC were similar across the motion profiles generated for this experiment.

Following these results, we sought to identify whether the underestimation was significantly non-zero. To that end, a linear mixed effects model was fitted with random effects of the subjects across the seven motion profiles, once for roll tilt and separately for pitch tilt. The results from this model showed an average −7.5% underestimation in roll that was not statistically significant (*t*(60) = −0.68, *p* = 0.50) and an average −33.2% significant underestimation in pitch (*t*(60) = −4.39, *p* < 0.0005). This can be visualized by the dotted horizontal lines in Figure 3: the mean intercept across the subjects and motion profiles.

**Figure 4:**
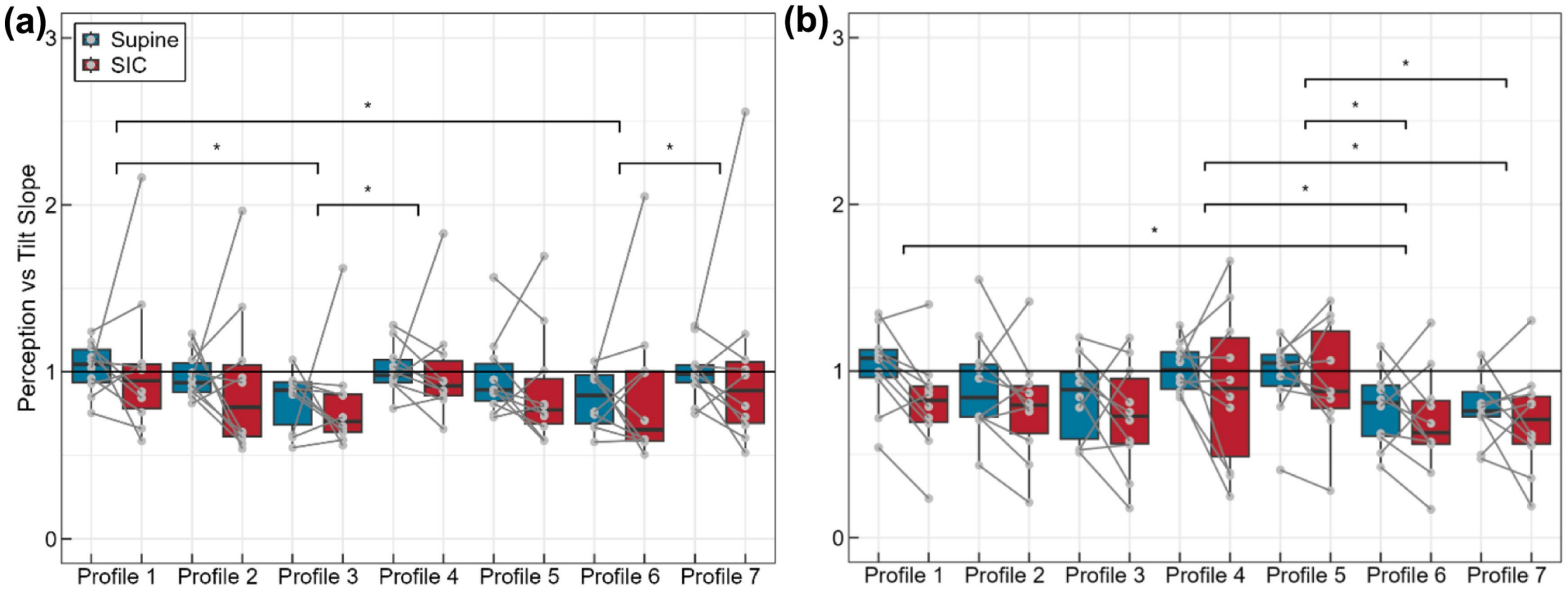
Perception Slope in roll **(a)** and pitch **(b)** across the unique profiles and SIC and Supine testing days. Significant differences are indicated by asterisks. The black line indicates unity. There are ten data points in each block.

An additional metric used to gauge the over- or underestimation between post-SIC and post-Supine perception was the perception slope. The effect of gravity condition (i.e., SIC or Supine) on this metric was investigated using a two-way ANOVA with repeated measures across profiles. While there was no significant effect of the gravity condition on the perception slope in roll (*F*(1, 9) = 0.008, *p* = 0.93) or pitch (*F*(1, 9) = 1.82, *p* = 0.21), there was a significant effect of the motion profile in roll (*F*(6, 54) = 3.21, *p* = 0.01) and pitch (*F*(6, 54) = 4.67, *p* = 0.0007). Post-hoc paired t-tests with a Holm correction revealed significant differences in the perception slope between various profiles in roll and pitch (Figure 4). The ANOVAs did not identify any significant interactions between the motion profiles and gravity conditions (*F*_roll_(6, 54) = 0.57, *p*_roll_ = 0.75; *F*_pitch_(6, 54) = 0.90, *p*_pitch_ = 0.5).

In pitch, while investigating broader models, a significant effect of ethnicity was also identified (*F*(1, 6) = 8.0, *p* = 0.03). While not an *a priori* hypothesis, for completeness, a linear mixed effect model was used to isolate the difference in perception slope between individuals who self-identified as White and non-Hispanic/Latino and those who did not, considering the random effects of each subject. The results showed that participants who identified as White and non-Hispanic/Latino had a significantly higher perception slope than those who did not (*t*(8) = 2.72, *p* = 0.026), indicating a greater response gain.

To investigate whether any of these differences could be due to the frequency content of the profiles, a two-way ANOVA was performed with repeated measures in the gravity condition and “frequency range” of the motion profiles. Here, the profiles were grouped by the following frequency regimes: static tilt (Profiles 4 and 5), low frequency (Profiles 2 and 6), and high frequency (Profiles 1, 3, and 7). In roll, this ANOVA did not identify any significant effects; however, in pitch, it did find a significant effect of frequency (*F*(2, 18) = 8.35, *p* = 0.003). Post-hoc pairwise t-tests with a Holm correction found that the perception slope for the static profiles was significantly higher than that for the low-frequency profiles (Δ = 0.17, *t* (19) = 3.55, *p* = 0.004) and high-frequency profiles (Δ = 0.15, *t* (19) = 3.91, *p* = 0.003), but there was no significant difference between the low- and high-frequency profiles. There was still no significant effect of the gravity condition or the interaction of the gravity condition with the frequency of motion profiles.

Since the goal of this study was to investigate tilt perception following SIC, motion sickness was monitored in case it interfered with perceptual reporting. Throughout the experiment, FMS scores did not exceed 10 out of 20. The maximum score reported following SIC averaged 1.9±2.67 (standard deviation) across subjects suggesting that some motion sickness was produced, but the motions were fairly well-tolerated from a motion sickness perspective. While previous studies have used this exact SIC protocol and centrifuge device to generate motion sickness (26, 27), it is likely that the less severe motion sickness reported during this experiment was due to the short duration of the motion profiles, completely passive motions, frequent breaks between trials, and complete darkness during motion (i.e., no visual information). It may also be due to our SIC protocol, which only reaches 2Gx for an hour. Motion sickness following SIC was found to be most severe following aggressive, active head tilts after being exposed to up to 3Gx for up to two hours (16, 20, 24), and previous studies have identified that the effects of SIC are stronger for higher force vectors and longer durations (20).

## Discussion

Here, we quantified how Sickness Induced by Centrifugation (SIC), a previously utilized sensorimotor analog for astronaut gravity transitions, impacted roll and pitch tilt perception. Using a within-subject design with two testing cohorts, we exposed 20 subjects to both 1Gx and 2Gx gravity environments before having them report their perception of tilt in a variety of static and dynamic motion profiles. In pitch, we found a systematic underestimation of tilt perception following the 2Gx gravity condition when compared to the 1Gx baseline, with the significant underestimation in pitch estimated as over four times larger than the non-significant underestimation in roll ( $\%\Delta_{\text{roll}}=-7.5\%,\%\Delta_{\text{pitch}}=-33.2\%$ ). While the direction of the misperceptions following SIC (i.e., underestimation) was similar between roll and pitch, we can speculate as to why the effect was much larger for pitch tilt perception and not statistically significant for roll by further considering the physical orientation of the otoliths.

The otoliths are comprised of two maculae: the utricle and saccule. The utricular plane is pitched approximately 30° up from the head level (37, 38) and is roughly level in roll, while the primary axis of the saccule is roughly perpendicular to the utricular plane. We acknowledge that the utricle and saccule are three-dimensional surfaces; thus, it is a simplification to consider only their primary planar orientations. Nonetheless, if the typical vestibular physiological head-fixed coordinates have the *x*-axis out of the nose, the *y*-axis out of the left ear (and the *z*-axis out of the top of the head), utricular plane axes could be defined with the utricular *x*-axis pitched up 30° from the head *x*-axis and the utricular and head y-axes remaining aligned. During SIC, the orientation of the hypergravity net force vector is applied in the head-centered negative *x*-axis (Supplementary Figure 1); thus, only the utricular *x*-axis and saccular maculae experience shear force (while the utricular *y*-axis does not experience shear force). Following SIC, subjects undergoing roll tilt only experience dynamic force (i.e., changing stimulation with the tilt of the chair) in the utricular *y*-axis (and slight changes in the saccular direction, which is less sensitive to roll tilt (39)). Thus, it may be expected that there would be a weaker effect on roll tilt perception. In contrast, during pitch tilt, the dynamic shear force is in the utricular *x*-axis (and saccular direction), both of which were abnormally stimulated by SIC. This may provide a basis for the physiological mechanism by which SIC has a larger impact on pitch tilt perception. Future studies could explore hypergravity centrifugation paradigms in which the direction of the net force is parallel to the pitch axis but perpendicular to the roll axis to evaluate this hypothesis.

The pitched-up orientation of the utricular plane might further be hypothesized to lead to differential effects of SIC on pitch tilt perception for tilts forward (nose down) vs. backward (nose up). For example, pitch tilt perception in hypergravity has been observed (40–42) not to be overestimated like in roll tilt, but instead to have an illusory perception of being pitched more nose up during pitch tilts forward, upright, and slightly backward. When the pitch is tilted backward by 30°, such that the utricular plane is perpendicular to the gravitational force vector, the pitch tilt perception is unaffected by hypergravity (41, 42). To evaluate this asymmetric effect for pitch tilt following SIC, we visualized pitch tilt perceptions separated out by whether the pitch tilt motions were “negative” or “positive” (Supplementary Figures 7, 8) but found similar trends, in which SIC causes underestimation relative to Supine, independent of pitch tilt direction (forward or backward starting tilt direction). Thus, we conclude that the pitched orientation of the utricular plane may contribute to our finding that pitch tilt perception is more impacted by SIC than roll tilt, but that the direction of pitch tilt (forward or backward) is similar.

While we did not identify the same underestimation following SIC in pitch using a perception slope metric, which looked at the linear relationship between tilt perception and actual tilt, it did identify significant effects of the different profiles on tilt reporting, as well as a significant effect of ethnicity on reporting. While, visually, it does appear that the perception slope following SIC tended to be lower than that following Supine, the metric is less of a direct measure of changes in perception comparing SIC to Supine than the percent difference measure. The perception slope metric focuses on over- or underestimation in comparison to the actual tilt profile. Instead of comparing perception in SIC to perception in Supine, this metric captures changes in perception relative to the actual tilt resulting from specific profiles that may or may not be affected by SIC. As such, it is useful in identifying how a participant’s tilt perception may be influenced by the motion profiles, even if it is not impacted by SIC.

Differences in perception across profiles are not unexpected. The semicircular canals within the vestibular system—responsible for sensing angular velocity—function as high-pass filters, meaning that high-frequency motions would be better sensed than low-frequency motions. The otoliths—which sense linear accelerations, including tilts with respect to gravity—are believed to be the organs primarily impacted by gravity transitions. Therefore, we might expect to see changes in perception during low-frequency motions, such as in Profiles 2 and 6, where there is more reliance on the otoliths, or in profiles with translations, such as Profiles 6 and 7, where the otoliths may conflate tilt and translation.

In roll, the perception slope tended to be lower in Profiles 3 and 6 (Figure 4a). Since Profile 3 was the tilt-only high-frequency profile, where it would be expected that both the semicircular canals and otoliths would be sensitive, it is possible that this underestimation was due to limits in subject SHH response during more rapid motions rather than changes in perception. Subjects may have been unable to rotate the bar to their perceived orientation before needing to move it again. Conversely, in Profile 6, containing both low-frequency motion and translation, an underestimation might be expected. In pitch, the perception slope is again smaller in Profile 3 (high-frequency tilt), Profile 6 (low-frequency tilt and translation), and Profile 7 (high-frequency tilt and translation). The underestimation in Profile 7 could be due to the presence of translation impacting otolith influence on the perception of tilt. Our frequency analysis did not identify any categorical variations in roll perception across the various frequency regimes but did identify differences in pitch tilt, where static profiles had categorically less underestimation than dynamic profiles.

The generalized change in pitch tilt perception found with the percent difference metric informs the use of SIC as a spaceflight analog for spatial disorientation and complements the use of SIC for other vestibular-impacted reflexes. Ocular counter-roll (OCR) is a vestibular-mediated reflex through which vestibular-sensed tilts result in counterrotations of the eye. Groen et al. (19) investigated the impact of SIC on ocular torsion during both dynamic and static roll tilts before and after SIC. For static tilts, where the reflex is dominated by the otoliths, they found a 10% decrease in ocular torsion (19). If this reduction was due to the impacts of SIC on the otoliths, it would be consistent with the tilt underestimation this study quantified: following SIC, a given head tilt stimulus induces less of a vestibular-driven response, whether reflexive or perceptual. However, spatial orientation processing and vestibulo-ocular reflexes likely involve different neural pathways that could be differentially impacted by SIC (43). Research investigating OCR following spaceflight has likewise found decreases in torsion immediately after returning from microgravity (44–47); however, these decays were paired with predominantly roll tilt overestimation (44), with minimal changes identified in pitch tilt perception (11). It is important to note that these results suggest that roll tilt perception is predominantly impacted by spaceflight only consider Shuttle missions (less than 2 weeks in microgravity) rather than longer-duration missions, where the impacts, including on pitch tilt perception, may be more profound (48). Here, we aimed to quantify roll and pitch tilt perception following SIC, an existing gravity transition sensorimotor analog. Future studies should directly compare post-SIC and post-spaceflight alterations in roll and pitch tilt perceptions.

The underestimation of pitch-tilt perception following SIC quantification likely contributes to other effects of SIC that have been previously observed, such as motion sickness and postural instability. Sensory conflict theory (49, 50)—the theory that posits that differences between expected and actual sensory information produce motion sickness—relies on a central expectation of how vestibular information is interpreted. Following SIC, the underestimation of tilt perception suggests a change in this expectation, which produces sensory conflicts and eventually motion sickness. Additionally, incorrect perceptions of tilt can lead to reduced or delayed postural adjustments during balance or mobility tasks, thereby elevating postural sway, which has been found to be more degraded in the anterior–posterior plane than in the mediolateral plane (28, 51). This further suggests that the pitch plane experiences the strongest effects of SIC in the Gx configuration.

### Limitations

The SIC analog is predominantly used to recreate the symptoms of gravity transitions, such as motion sickness, postural instability, and gait destabilization. This is the first quantified application of SIC specifically for gravity transition-induced tilt misperceptions and spatial disorientation. As such, it is critical to discuss the potential ways in which this analogy may be an imperfect comparison. First, the adaptation process in SIC differs from that in spaceflight in terms of the time course. When astronauts first enter microgravity, symptoms of Space Motion Sickness can persist for a few days as their vestibular system adapts to the new gravity environment (52, 53). An important aspect of this adaptation includes the head movements of the astronaut in microgravity, by which they provide information for the vestibular system to adapt. A recent study by Kravets et al. found no evidence of changes in spatial orientation perception over the time course of one hour of hypergravity with head tilts, which suggests that central reinterpretation requires longer time scales (54). Using SIC, we assume that some aspects of this adaptation may occur in one hour of centrifugation with the subject’s head fixed in place; however, it is unlikely to drive any central reinterpretations of canal and otolith integration, including models that disambiguate tilt and translation—one such mechanism that has been posed for neuro-vestibular adaptation to spaceflight (12). Rather, it is more likely that any adaptation effects occur due to changes at the level of the vestibular periphery. Specifically, there may be a decay in otolith firing rates following prolonged centrifugation (55). This may result in a change in the central expectation of the baseline firing rate rather than a reinterpretation of the canal and otolith cues (12, 56, 57).

Next, there is also the question of the direction and magnitude of the gravity transition involved in SIC. Previous hypogravity (8, 9) and hypergravity (7) studies have identified a “tilt gain” phenomenon, wherein tilt perception following a transition to a smaller magnitude of gravity results in tilt underestimations, and a larger magnitude of gravity results in tilt overestimations (8, 58). Assuming that adaptation to hypergravity has occurred while in 2Gx during SIC, upon transition back to 1G, we might expect underestimation; however, this does not capture the effects of pure microgravity—a singularity given the complete absence of gravitational cues—and so it may not be a direct comparison to the transition from Earth to microgravity, even though the magnitude of change and direction of change are the same.

Additionally, for a lunar landing mission where astronauts are in transit for at least three days, it is unclear how much the time spent in microgravity will impact their perception during a lunar landing. It is unknown whether the dominant transition will be from 1G on Earth to 1/6G on the Moon—which would theoretically result in tilt underestimation—or from microgravity during transit to lunar gravity, which may produce a tilt overestimation. Given these uncertainties, SIC should only be considered a tool for understanding the effects of gravity transitions on tilt perception, rather than as a one-to-one analog for any specific mission.

Beyond the paradigms leveraged, the results are limited by the psychophysical task subjects performed. In both roll and pitch tilt, the SHH task utilized a physical interface for indicating perceptions of tilt. Therefore, it is not a purely vestibular task but includes aspects of the proprioceptive and tactile systems that may impact results. In pitch, this was especially evident given the asymmetrical nature of the reporting plate. While the plate was leveled at the beginning of each trial, subjects resting their arms on the armrests tended to drift toward a more comfortable neutral position during the trials. These effects were identified during data correction as a strong negative bias in the pitch direction (corresponding to participants tilting the plate backward more than the chair was pitched forward for both SIC and Supine conditions).

Furthermore, given that the pitch reporting plate was mounted on the right side of the chair, the subjects were required to use their right hand, regardless of handedness. Although it was possible to swap the sides of the plate, in pilot testing with a left-handed lab member, the task was deemed easy enough with their non-dominant hand. Additionally, the only left-handed subject in the pitch cohort seemed comfortable with the task during the practice trials and did not produce any data that needed to be removed due to non-perceptual reporting.

In addition to the potential proprioceptive impacts, there may have also been somatosensory cues and other graviceptor cues contributing to tilt perception beyond vestibular changes from SIC. Much like the otoliths, the somatosensory system consists of graviceptors that track the direction and magnitude of the net gravito-inertial force vector and may play a role in tilt perception. Since both systems are sensitive to changes in gravity during full-body tilts, it is difficult to separate the effects of either, and instead, we acknowledge that the results we found may also be influenced by changes in somatosensory expectations as well.

### Future work

This study represents the first instance of quantifying changes in tilt perception following SIC exposure. Using SIC as a spaceflight analog, this empirical data can be utilized to update models of orientation perception in a spaceflight environment. Specifically, we are interested in using this data to retune the Observer Model (59, 60). The Observer Model is a computation that compares simulated vestibular outputs from physical motions with expected vestibular outputs from a central nervous system approximation to produce orientation perception. Presently, it is well-tuned for identifying illusions that arise from terrestrial aviation; however, the data from this study can be used to retune the model for a more relevant spaceflight environment. With a retuned model, the system can be adapted to monitor the orientation perception during lunar or Martian landing operations.

It would also be interesting to consider how different spaceflight analogs compare to SIC when considering their impact on tilt perception and sensorimotor performance. A previous study by Moore et al. successfully utilized galvanic vestibular stimulation (GVS) with a pseudorandom sum-of-sines binaural signal to produce off-nominal simulated Shuttle landings (61) and impact locomotion (62). Additionally, they attempted to use a GVS signal that was head-coupled with yaw velocity and heave acceleration to impact locomotion but only identified deterioration in obstacle course performance, not general treadmill walking, where the head motion is minimal (62). Allred et al. further built on the idea of head-coupled binaural GVS to investigate which coupling schemes produced the best amplification or attenuation of a tilt profile (29) and found that angle-coupled GVS may be a promising route for intentionally modifying tilt perception as would be relevant for a spaceflight analog. The effects of GVS are most significant in the roll plane, which suggests that it may even complement the effects of SIC, which this study has shown to be stronger in pitch.

GVS has also been used in tandem with visual disorientation prism goggles and a weighted vest to recreate sensorimotor decrements relevant to spaceflight. This sensorimotor disorientation analog (63) has been compared to the SIC paradigm (using 3Gx for 60 min) to reveal similarly impacted performance in functional tasks. In general, both analogs negatively impacted fall recovery performance, a four-square step task, and an obstacle-turning task (64).

Furthermore, other analogs, such as the Wheelchair Head-Immobilization Paradigm (WHIP), have also shown promise in impacting tilt perception and spatial orientation (57). In this analog, subjects lay on their sides on a platform mounted to a motorized wheelchair, with their heads restrained to prevent any head tilt relative to gravity. Following 12 hours of this protocol, the participants reported illusory sensations of motion and experienced decreased balance and functional mobility performance. Rather than targeting the otoliths, as with SIC, this protocol aims to decouple cues from the semicircular canals and otoliths, as is relevant for spaceflight. Future studies should directly compare the effects of these various sensorimotor spaceflight analogs on tilt perception, as well as functional performance.

## Data Availability

The datasets presented in this study can be found in online repositories. The names of the repository/repositories and accession number(s) can be found at: https://osf.io/4n7wr/?view_only=f9b7a833af3347aeabca6f811707a313.
