# Supplementary material for: Impact of sickness induced by centrifugation on tilt perception
Source: Front Neurol. 2025 Aug 13;16:1628938. doi: 10.3389/fneur.2025.1628938 (PMC12380784; doi:10.3389/fneur.2025.1628938)
Supplement: Supplementary file 1 [file Supplementary_file_1.docx]

Supplementary Figure 1. The motion devices used to determine the impact of SIC on tilt perception. The centrifuge (a and b) rotated at 24 RPM to generate a net 2Gs at the head while the sled (c and d) produced roll tilts and lateral translations or pitch tilts and fore-aft translations (shown in roll configuration).


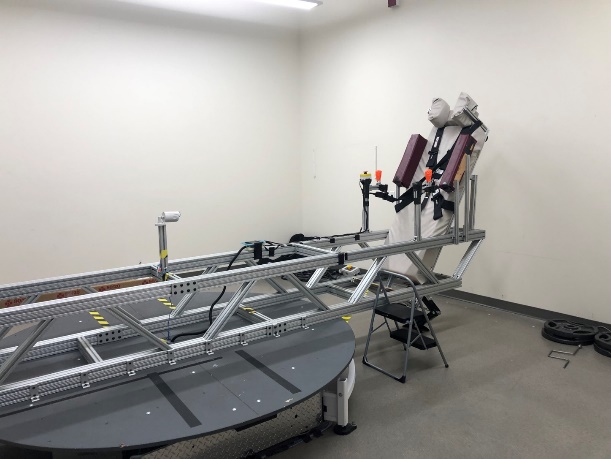

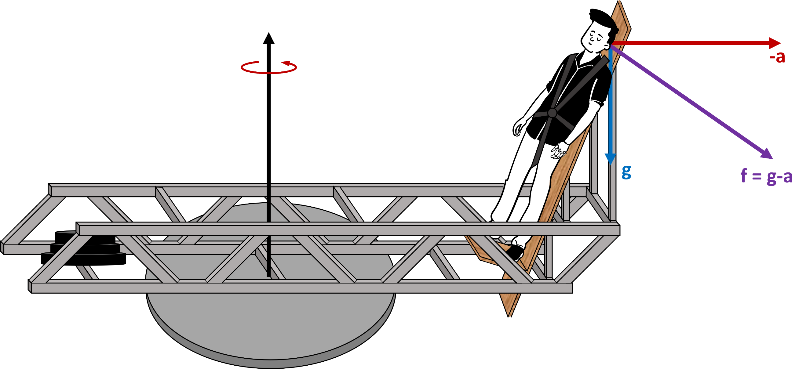

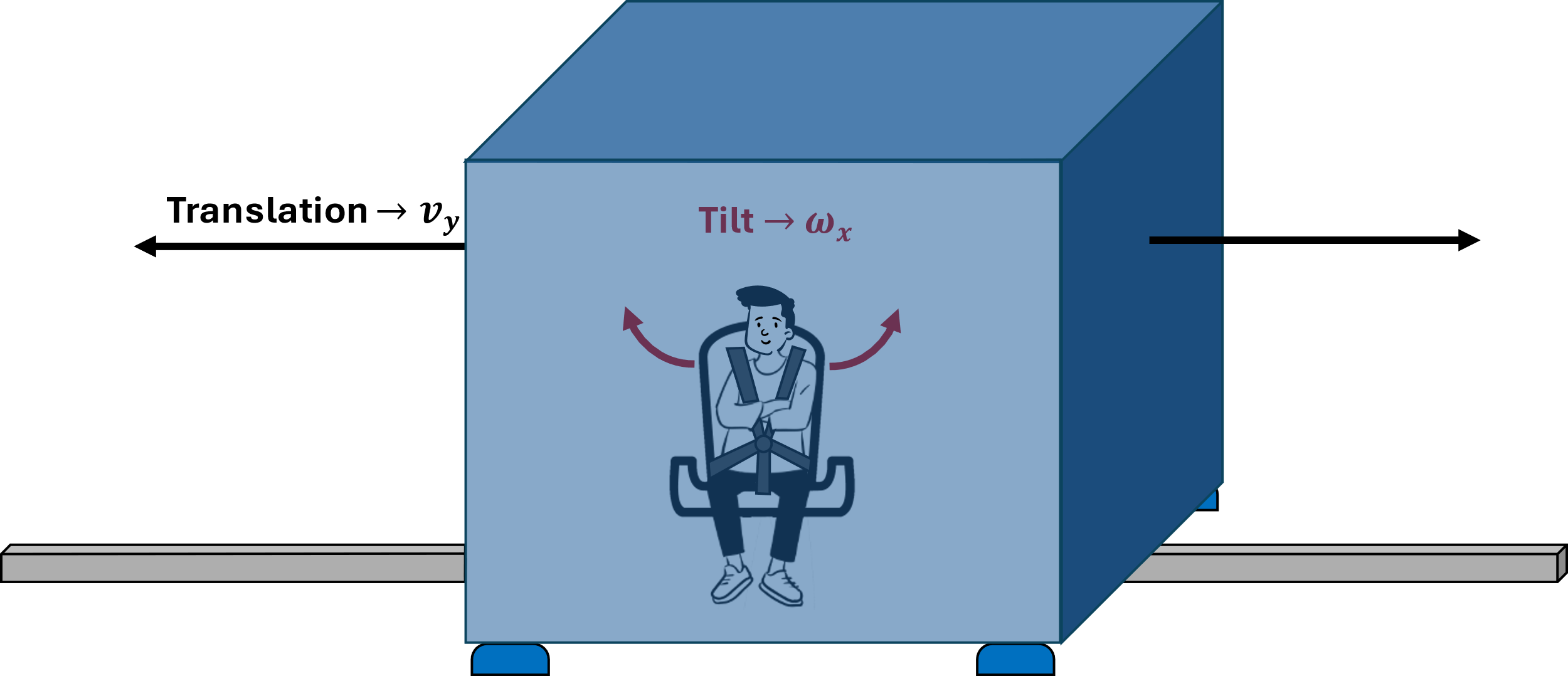

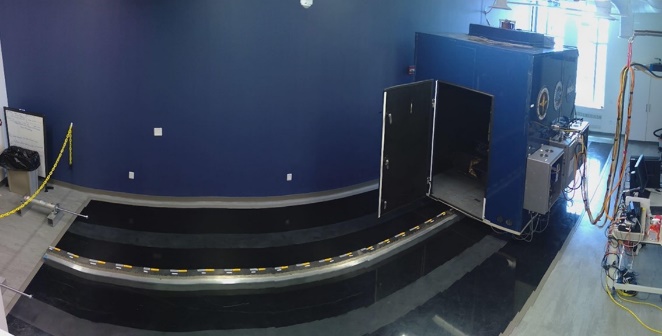


**(c)**

**(a)**

**(b)**

**(d)**

Supplementary Figure 2. Data corrections in roll tilt. The bias correction(a) is only measured for the dynamic profiles (black symbols) but are shown here for the two static profiles as well. The median time shift for each subject is shown in (b) across both testing days. The larger circle indicates two subjects with the same time shifts. Gain is shown in (c) on a logarithmic scale.

**(a)**

**(b)**

**(c)**

Supplementary Figure 3. Data corrections in pitch. The bias correction(a) is only measured for the dynamic profiles (black symbols) but are shown here for the two static profiles as well. The shapes indicate unique subjects and are different subjects then those in Roll cohort. The median time shift for each subject is shown in (b) across both testing days. Gain is shown in (c) on a logarithmic scale.

**(a)**

**(b)**

**(c)**

Supplementary Figure 4. Example of the perception slope metric. The blue data points are the SHH points plotted against the actual tilt at every time point. The blue solid line is the best linear fit of these data points while the red solid line is unity.

Supplementary Figure 5. Roll tilt perception over time. The SIC1 lines the average perception after the first two trials across all subjects. The SIC2 line is the average perception after the last two trials across all subjects. Including the standard error in the shaded regions, there is no recovery from SIC1 to SIC2.

Supplementary Figure 6. Pitch tilt perception over time. The SIC1 lines the average perception after the first two trials across all subjects. The SIC2 line is the average perception after the last two trials across all subjects. Including the standard error in the shaded regions, there is no recovery from SIC1 to SIC2.

Supplementary Figure 7. Perception of pitch tilt during profiles starting in the “positive” direction. The testing days are designated with blue for Supine and red for SIC with respective shaded regions indicating standard error.

Supplementary Figure 8. Perception of pitch tilt during profiles starting in the “negative” direction. The testing days are designated with blue for Supine and red for SIC with respective shaded regions indicating standard error.
